# Digital MULTIMAP: a standardization of objects and actions naming task in a french population

**DOI:** 10.1007/s00701-026-06927-y

**Published:** 2026-06-04

**Authors:** Marion Barberis, Anouk Malewski, Franck Tarpin-Bernard, Ileana Quiñones, Manuel Carreiras, Isabelle Poisson, Emmanuel Mandonnet

**Affiliations:** 1 Department of Neurosurgery, Lariboisière Hospital, AP-HP, 2 Rue Ambroise Paré, 75010 Paris, France; 2 Scientific Brain Training (SBT) Humans Matter, Lyon, France; 3 Biogipuzkoa Health Research Institute, Pº Dr. Beguiristain s/n, 20014 Donostia – San Sebastián, Gipuzkoa Spain; 4 Basque Center On Cognition, Brain and Language, Paseo de Mikeletegi 69, 20009 Donostia – San Sebastián, Gipuzkoa Spain; 5 Ikerbasque, Basque Foundation for Science, Plaza Euskadi 5, 48009 Bilbao, Bizkaia Spain; 6 University of the Basque Country, UPV/EHU, 48940 Bilbao, Spain; 7 Frontlab, CNRS UMR 7225, INSERM U1127, Paris Brain Institute (ICM), Paris, France; 8 Université de Paris Cité, Paris, France

**Keywords:** Picture naming task, Low-grade glioma, Awake surgery, Assessment, French standardization

## Abstract

**Purpose:**

Picture naming is a widely used task for perioperative language assessment and brain mapping in awake surgery for brain tumors. Although there is a consensus on this task between centers performing this type of surgery, the tests used vary from one team to another. The MULTIMAP picture naming task, initially developed in 2021 by the Basque Center on Cognition, Brain and Language, aimed to enhance the perioperative assessment of lexical access of both nouns and verbs in awake surgery patients and to facilitate standardized protocols for international multicenter studies. This study presents the French adaptation, digitization, and standardization of extraoperative MULTIMAP.

**Methods:**

The tool was standardized on a sample of 416 healthy subjects recruited from the French population, whose performances (score and time) were statistically analyzed. Of the 100 items tested, we retained the 80 (40 objects, 40 actions) that showed the highest naming accuracy and were balanced between nouns and verbs for main psycholinguistic variables.

**Results:**

Gender had no effect on performance, the level of education had a significant effect for the action naming task only, and age for both tasks, allowing to determine percentiles according to these variables. Performances in object and action naming were correlated; however, a significant but minimal difference between the average score and time between tasks was found.

**Conclusion:**

The digitized French version of MULTIMAP is a promising tool, that awaits further validation in patients with acquired brain lesions, especially in the context of brain tumor awake surgery.

**Supplementary Information:**

The online version contains supplementary material available at 10.1007/s00701-026-06927-y.

## Introduction

Over the past two decades, awake surgery with intraoperative cortical and subcortical mapping became a more and more popular methodology for achieving brain tumor maximal safe resection. During the surgery, the neurosurgeon disrupts specific functional areas around the tumor by applying cortico-subcortical electrical stimulation, while the patient performs language or cognitive tasks supervised by a speech therapist, clinical linguist, or neuropsychologist [35]. Errors or changes in the patient's response enable the neurosurgeon to establish a functional dynamic map of the brain structures involved, hence to stop the resection when the optimal onco-functional balance is thought to be reached [7, 8]. In this context, awake surgery teams have developed their own battery of cognitive and language tasks, both for extra- and intraoperative monitoring [35].

Picture naming (of objects and/or actions) is a cornerstone of the neuropsychology of language, as it provides highly informative insights at every level of analysis—cognitive, neuroanatomical, and clinical. First, the task taps into all subprocesses of the standard cognitive model of single word processing (see for example [14] and [34]), i.e. conceptual semantic, lexical, phonological, and articulatory levels. Moreover, it also probes some basics of morphosyntax (e.g., determinants for nouns, conjugation for verbs). Second, at the neuroanatomical level, in-depth analysis of the type of errors in picture naming – e.g., semantic versus phonological paraphasia—allows to map both the ventral and dorsal streams, hence covering a large part of the entire language network. (see for example [9] and [13]). Last but not least, despite picture naming does not directly evaluate multifaceted language abilities such as spontaneous speech (also named connected speech) [38], which are essential to daily communication, it has been reported that better naming correlates with better spontaneous speech in aphasic patients [3, 21]. In line with these results, very high rates of return to work were observed in a large cohorts of patients with IDH-mutated glioma resected under electrical mapping with picture naming (and reading in posterior temporo-occipital areas) [27], demonstrating the clinical and ecological value of the task, at least in the long term (non-tested language functions might be of course impaired in the acute postoperative period, but lately recovered by plasticity-mediated reshaping of brain networks). For all these reasons, picture naming is, of the numerous tasks included at all stages of language assessment of awake surgery patients (preoperative, intraoperative, postoperative, follow-ups), the one on which there is the greatest consensus across centers. Used in all European centers [19] and listed in systematic literature reviews [4, 20], picture naming is considered as a must-have task in the armamentarium of the speech therapist/clinical linguist/neuropsychologist, both extra- and intraoperatively [25]. Accordingly, many teams around the world have developed (or adapted from another language) and standardized their own picture naming test, including but not limited to [2, 6, 28, 30, 31].

In parallel, researchers in psycholinguistics have created databases of the main psycholinguistics variables (such as frequency, number of syllables, familiarity, age of acquisition, …) for selected sets of words. As these variables are known to influence both accuracy and time response of items in healthy subjects, their knowledge should also enable to refine the evaluation of naming abilities in aphasic patients, by performing item-level analysis. However, such item-level approaches are not commonly used in clinical context (see however [31, 40]). Still, controlling these variables can be useful when there is a need to render two tasks “comparable in difficulty”, such as naming nouns versus verbs, or when transposing a test from one language to another (see [10] for challenges to be solved when adapting a task from one language to another one). This double constraint motivated the MULTIMAP initiative [11], with the specific goal of generating bilingual lists of objects and action pictures for *intraoperative* mapping of naming, controlling the main psycholinguistic variables between nouns and verbs within each language and across languages. The underlying objective was to enhance the interpretability of intraoperative monitoring: for example, if the patient is making more errors for verbs rather than nouns, we do not want this to be attributed to an unbalanced distribution of the frequency variable between the two sets of items.

Here, we propose to further elaborate on the MULTIMAP material, but with a slightly different perspective. Regarding intraoperative mapping, we adhere to the “causal” paradigm: because we exclude from the intraoperative list all items erroneously named preoperatively, we interpret any error under stimulation as causally generated from the disturbance induced by the stimulation, whatever the item difficulty (albeit to ensure with confidence that the error was indeed caused by the stimulation and not some attentional fluctuation or any other interfering event, at least 2 failures out of 3 trials under stimulation are required, with correct responses on intermixed trials without stimulation). Hence, our aim was rather to develop a well-standardized French digital picture naming task for longitudinal *extra-operative* assessment, which could be easily adapted in other languages. These longitudinal evaluations constitute the necessary step to clear the line between research and clinics in awake surgery: introducing a new task is justified only if there is a risk that patients suffer long-term deficits [18]. To the best of our knowledge, while it has been clearly demonstrated that brain networks sustaining objects and actions naming are at least partly dissociable [33, 36, 37] (implying a risk of dissociated acute postop deficits), it remains unknown how the two networks can compensate each other by plasticity in the long run. Such a picture naming test would greatly facilitate international multicentric longitudinal data collection to address this important question. Moreover, for research, reliable standardization of cognitive tasks is essential for inferring individual graph-based cognitive networks (see [12]).

In France, most centers use the DO80 picture naming task [5]. However, this test suffers some limitations: designed 30 years ago for language deficits induced by strokes, it contains some old-fashioned graphics (e.g., the corded phone) and a few others confusing (e.g., the accordion); its norms are based on a small healthy-control population (106 subjects); it does not evaluate lexical acces to verbs; it exists only as a paper-and-pencil material.

Our objectives were thus:

- To select from the MULTIMAP dataset a list of 40 nouns and 40 verbs (80 pictures), with main psycholinguistic variables controlled between nouns and verbs,
- To design a user-friendly digital app to administrate the task, allowing to assess both accuracy and time responses,
- To standardize the test in a large sample of healthy subjects, that is to explore the influence on performance of age, education, and sex and to provide percentiles norms according to these variables

## Materials and methods

### Material

**Fig. 1:**
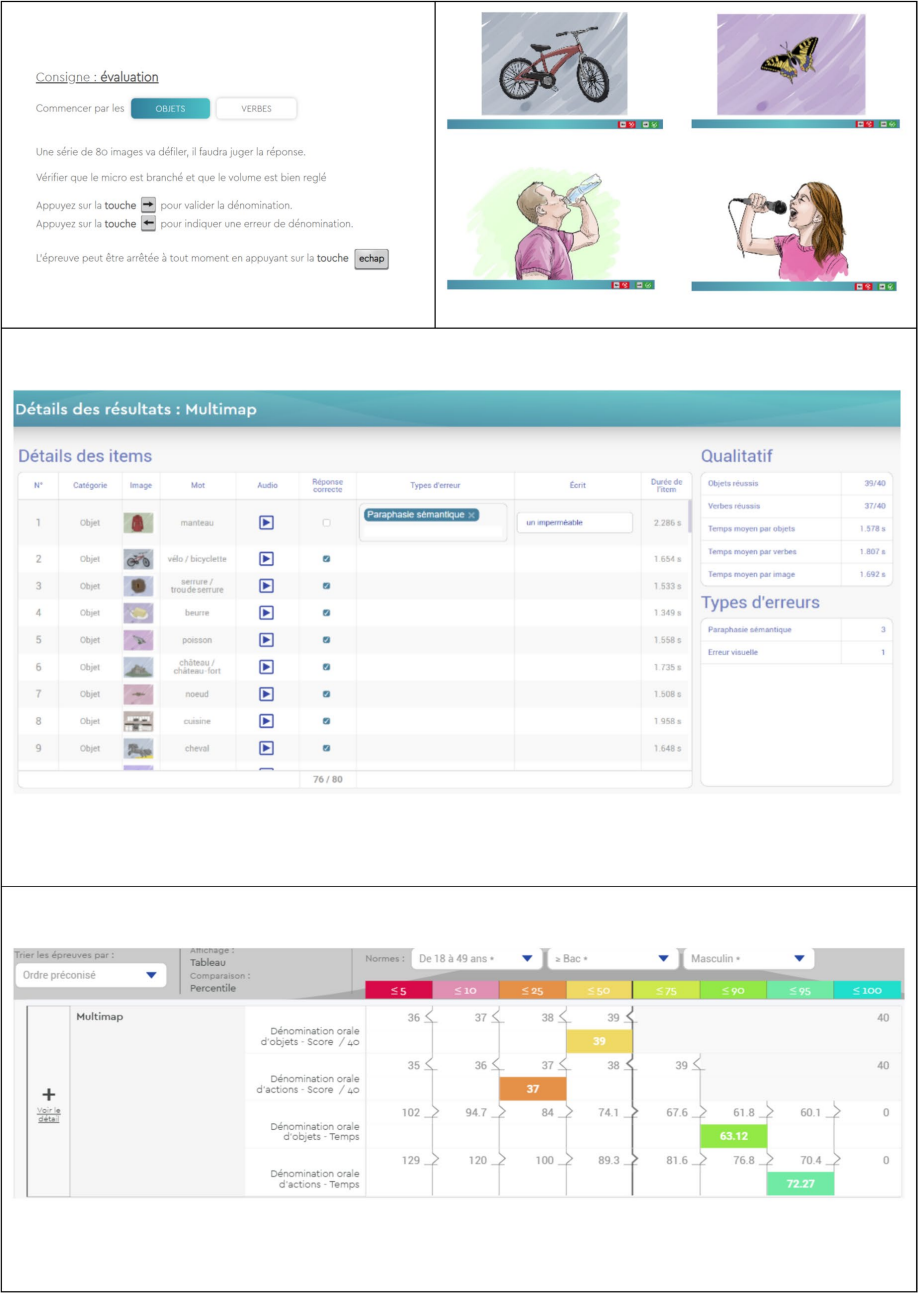
Digitized French MULTIMAP: instructions, stimuli, and results overview**.** *Note.* Task instructions (upper left panel); two objects and two actions images (upper right panel); replay and results processing interface with detailed information on stimulus duration, type of error, (central panel); results summary displaying scores and times relative to the standardized norms (bottom panel)

The digitized French version of MULTIMAP was adapted from the MULTIMAP picture naming test [11], whose authors provide 218 royalty-free color images with controlled psycholinguistic variables in different languages. The team from the Basque Center for Cognition, Brain and Language validated several monolingual and bilingual lists of items with the aim of creating an international tool for assessing intraoperatively lexical retrieval. We used the two French monolingual lists of 40 nouns and 40 verbs (with frequency, number of letters and H-index controlled between nouns and verbs) and added four items for task instruction (two images of objects, two images of actions). Due to a cultural gap, some items were likely to have a very low picture recognition rate in the French population. We thus randomly selected ten additional nouns and ten verbs from the bank of images. The set of images in our study therefore included 104 items: 52 nouns (two example images and 50 test images) and 52 verbs (two example images and 50 test images). The test was then digitized in collaboration with HappyNeuron (https://www.happyneuron.fr/), a French platform dedicated to cognitive assessment and rehabilitation, in a version enabling longitudinal assessments in a user-friendly interface (see Fig. 1). The software is a web-based application, allowing its use on a wide range of operating systems. The final selection of 40 nouns and 40 verbs was made in a such a way that: i. name agreement, H-index, number of letters, and frequency were controlled between nouns and verbs; ii. the histogram of scores did not show any ceiling effects, for both nouns and verbs. Number of letters and frequency values were retrieved from the French openlexicon database (https://openlexicon.fr/), while name agreement and H-index values were taken from [11].

The evaluation was carried out in real time by the examiner, by typing the green (good response) or red (bad response) virtual button at the bottom of the screen tablet, thus measuring a time response for each item. Total times and scores were automatically calculated at the end of the task. As subject's responses were audio recorded, the audio recording could be replayed to correct any scoring errors, to transcribe unexpected responses, and to qualify the type of language error if necessary (anomia, phonological/semantic paraphasia, syntactic error, etc.).

### Subjects

A total of 416 healthy control subjects aged from 18 to 95 years (mean age: 49.7) were enrolled between June 2021 and January 2022. Inclusion criteria were: being over 18 years of age; having French as mother tongue or having completed all of their schooling in France; having no cognitive deficit (score ≥ 26/30 at the Montreal Cognitive Assessement test 8.1, [26]). Exclusion criteria were: having significant visual impairment despite wearing glasses, screened with the Parinaud scale, a reading test commonly used by French ophthalmologists; having a history of language development disorder, cardiovascular, cognitive, neurological and psychiatric pathologies; having a dependence on a toxic substance or alcohol. As shown in Table 1, participants were recruited according to three demographic characteristics: gender (238 women/178 men); four age groups (18–29 years; 30–49 years; 50–69 years; 70 years and over); three levels of education, in reference to the baccalauréat (Bac), the French High School Diploma (< Bac = no Bac; Bac-Bac + 3 = Bac to three years of higher education after Bac; > Bac + 3 = more than three years of higher education after bac, corresponding respectively to: no High School Diploma; High School Diploma to Bachelor’s degree; Master’s degree and higher).

**Table 1:** Population’s characteristics

	< Bac ^a^		Bac-Bac + 3 ^b^		> Bac + 3 ^c^		
	Women	Men	Women	Men	Women	Men	n (%)
Age 18–29	16	15	18	16	23	16	104 (25%)
Age 30–49	17	14	18	15	21	18	103 (24.8%)
Age 50–69	22	18	22	11	20	16	109 (26.2%)
Age ≥ 70	20	12	20	11	21	16	100 (24%)
n (%)	134 (32.2%)		131 (31.5%)		151 (36.3%)		416 (100%)

^a^no French High School diploma;

^b^French High School diploma to Bachelor’s degree;

^c^Master’s degree and higher diploma

All participants were informed of the objective of the study via an information documentation explained by the examiner, and gave their written informed consent that could be revoked at any time. The study was approved on June 17th 2021 by the Commission Nationale de l’Informatique et des Libertés, in accordance with the General Data Protection Regulation (Data Protection Officer reference register: 2021–131).

### Study protocol

Seven speech-therapist students administered the inclusion questionnaire, the Montreal Cognitive Assessement test 8.1 [26], the visual screening test, and the digitized French MULTIMAP test in a quiet, distraction-free, and well-lit place. The students received specific training for administering and scoring the digitized French MULTIMAP to harmonize the data collection and minimize the inter-rater differences. Participants always started with the object naming task and continued with the action naming task. The images of the 50 nouns and the 50 verbs were always presented in the same order. Before each task, two examples were presented. Subjects had to give the noun preceded by an article for the objects, and a finite verb with third person singular for the actions. If the subject failed the examples, the expected response was given by the examiner; if the subject gave a response with expansion (noun followed by an adjective, or verb followed by a direct object), the examiner specified to strictly name only the object or action, without any added word. After the examples, the examiner had to rate the subject's answers (correct/incorrect) in real time using the keyboard arrows of their laptop or buttons on the touch screen of their tablet. Responses were considered as correct only if they matched the target word. Each part of the task was timed. The responses were recorded so that they could be listened again at the end of the task to correct any rating errors, to transcribe unexpected responses, and to qualify the type of language errors, if needed. Ultimately, the accuracy or inaccuracy of some unexpected responses was discussed and decided by group consensus.

### Statistics

Statistical analyses of the demographic effects on the scores of the digitized French MULTIMAP were performed with Jamovi 2.2.5. Normality of data distributions was tested according to the Shapiro–Wilk test. Non-parametric tests of correlation (Kendall test), comparison (Wilcoxon signed rank test), and variance (Kruskal–Wallis test), as well as post-hoc analyses (Dwass-Steel-Critchlow-Fligner test) were performed. Descriptive statistics (mean, standard deviation (SD), minimum, maximum, if data were normally distributed or percentile performance distribution if not) were calculated for each task, for the entire population, and for the significant subgroups. Comparison of psycholinguistic variables between nouns and verbs items was performed with Student* t* test. For all analysis, significant threshold was set at *p* = 0.05.

## Results

### Selection of 40 nouns and 40 verbs

The average reaction time and the average naming accuracy (or success rate) were calculated for each of the 100 test items (see Supplementary Table 1). For some pictures, we decided to accept alternative responses when these were given very frequently (see Supplementary Table 2 & 3). We removed items where the picture was difficult to recognize due to visual ambiguity (for example, the towel was very frequently confused with a wallet). We selected the 40 verbs with the highest naming accuracy. We then selected nouns items in such a way that name agreement, H-index, frequency and number of letters were controlled between nouns and verbs (see Supplementary Table 1).

The average name agreement was 96% (SD = 3.99) for the nouns and 94.2% (SD = 6) for the verbs (*p* = 0.16). The average H-index was 0.22 (SD = 0.22) for the nouns and 0.36 (SD = 0.39) for the verbs (*p* = 0.06). The average frequency was 60 (SD = 86) for the nouns and 44 (SD = 40) for verbs (*p* = 0.36). The average number of letters was 6.3 (SD = 1.4) for the nouns and 6.4 (SD = 1.1) for verbs (*p* = 0.66). Hence, the main psycholinguistic variables were balanced between nouns and verbs.

### Scores, response times: correlation within and between the tasks

#### Item-level analysis

The average success rate per item was 93.3% (SD = 8.6%) for nouns and 91.7% (SD = 7.8%) for verbs (Wilcoxon rank signed test, p < 0.001). The average response time per item was 2.2 s (SD = 0.5 s) for the nouns and 2.66 s (SD = 0.6 s) for the verbs (Wilcoxon rank signed test, p < 0.001). Average response times were lower than 4 s for all items except one (“construire”, (build), 4.2 s).

#### Task-level analysis

**Fig. 2:**
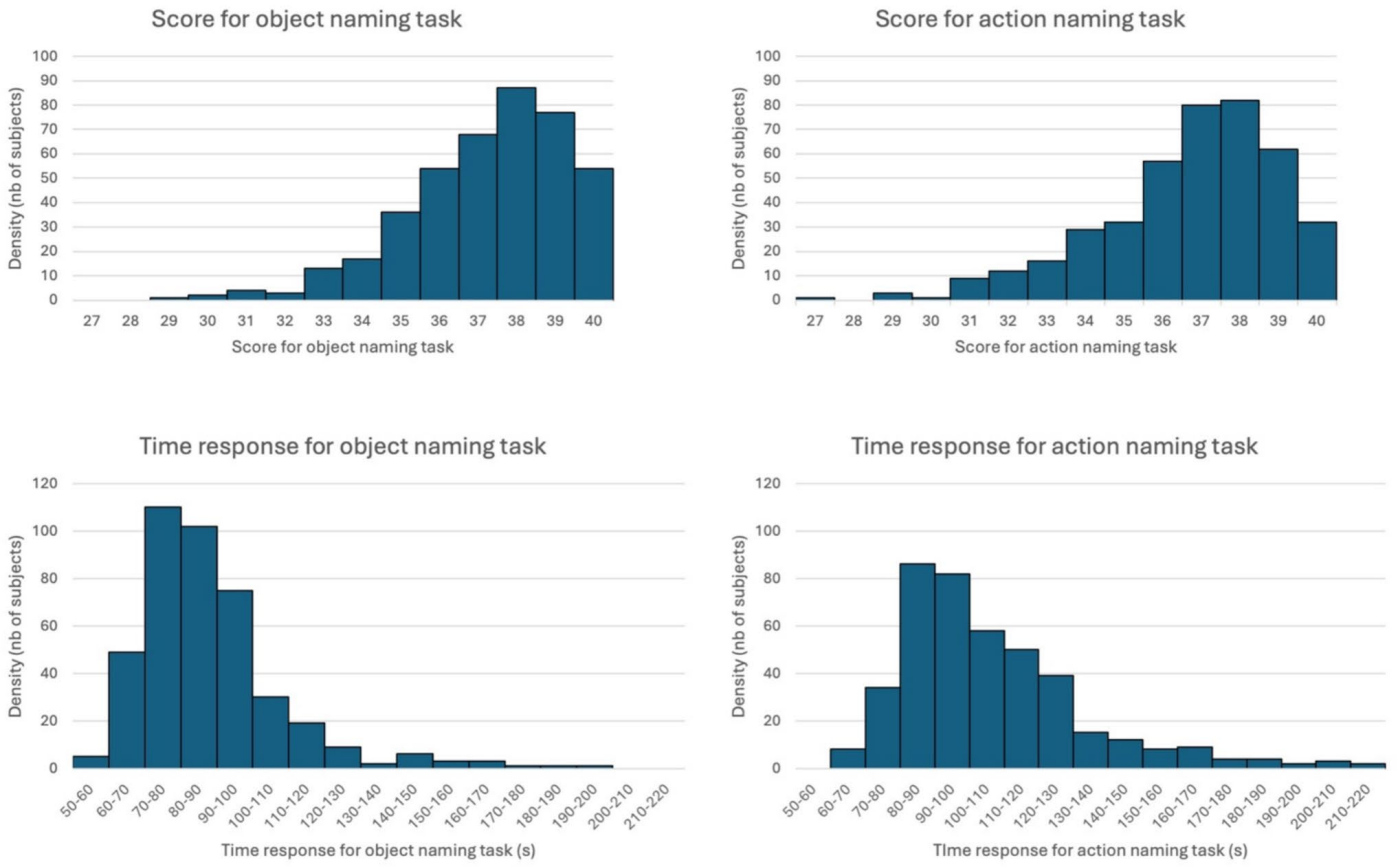
Distribution of scores and response times in object naming and action naming tasks

Figure 2 shows the distribution of scores and response times for both tasks. The average score was 37.3 (median = 38, SD = 2) for the nouns and 36.7 (median = 37, SD = 2.3) for the verbs (Wilcoxon rank signed test, p < 0.001). The average total time was 88 s (SD = 20 s) for nouns and 106 s (SD = 27 s) for verbs.

Significant correlations were found within and between each task, as presented in Table 2. Subjects who were faster to name objects were also faster to name verbs. Moreover, the faster the naming, the higher the scores.

**Table 2:** Correlation between scores and times

	Kendall tau-b			
	Score nouns	Score verbs	Time nouns (s)	Time verbs (s)
Score nouns	—			
Score verbs	0.347 ***	—		
Time nouns (s)	−0.320***	−0.173***	—	
Time verbs (s)	−0.252 ***	−0.263***	0.596 ***	—

***p < 0.001

### Effects of age, education, and gender on score and total response time

Among the three subjects' characteristics, age had the strongest effect on performance (Table 3). In each task, regarding the total response time, subjects aged 50 to 69 years were slower than younger (18–29 years and 30–49 years) but faster than older (≥ 70 years). For the action naming task, subjects aged 50 to 69 years had scores significantly lower than younger subjects (18–29 years and 30–49 years), but higher than older subjects (≥ 70 years). For the object naming task, subjects over 70 years had significantly lower scores than the rest of the population. In both tasks, there was no significant difference in score or total response time between the two youngest groups of subjects (18–29 years and 30–49 years).

**Table 3:** Effects of age, education, and gender on scores and response times in each task

		Objects		Actions	
		Score *p*	Total time *p*	Score *p*	Total time *p*
Age		< 0.001***	< 0.001***	< 0.001***	< 0.001***
18–29	30–49	0.966	0.596	0.960	0.979
18–29	50–69	0.206	< 0.001***	0.002*	< 0.001***
18–29	≥ 70	< 0.001***	< 0.001**	< 0.001***	< 0.001***
30–49	50–69	0.478	0.011*	< 0.001***	< 0.001***
30–49	≥ 70	< 0.001***	< 0.001***	< 0.001***	< 0.001***
50–69	≥ 70	0.017*	< 0.001***	< 0.001***	< 0.001***
Education		0.742	0.130	< 0.001***	0.035*
< Bac	Bac-Bac + 3	0.995	0.127	< 0.001***	0.024*
< Bac	> Bac + 3	0.774	0.367	< 0.001***	0.268
Bac-Bac + 3	> Bac + 3	0.802	0.705	0.957	0.567
Gender		0.845	0.298	0.545	0.367

* *p* < 0.05, ** *p* < 0.01, *** *p* < 0.001

The level of education had no effect on performance in the object naming task; however, it significantly influenced both accuracy and total response time in the action naming task. Subjects with the lowest level of education (< bac) showed lower accuracy and longer response times compared to those in the bac-bac + 3 and > bac + 3 groups, which performed similarly. Finally, gender had absolutely no effect on scores or response times in either task.

### Percentiles adjusted for age and educational level

Analysis of the effects of demographic characteristics on scores and response times identified the relevant categories: three age groups (18–49 years; 50–69 years; 70 years and over) and two educational level groups (< bac; ≥ bac). The norms therefore include six groups for the verb naming task: 18–49 years, < bac (*n* = 62); 18–49 years, ≥ bac (*n* = 145); 50–69 years < bac (*n* = 40); 50–69 years, ≥ bac (*n* = 69); 70 years and over, < bac (*n* = 32); 70 years and over, ≥ bac (*n* = 68). As no effect of level of education was found in the object naming, the norms therefore comprise three groups for the object naming task: 18–49 years (*n* = 207); 50–69 years (*n* = 109); 70 years and over (*n* = 100). The normative data for the entire population (*n* = 416) can be found in the Supplementary Table 4 & 5 for nouns and verbs, respectively.

## Discussion

The aim of this study was to develop and standardize a digitized French version of MULTIMAP, a picture naming test specifically designed for the assessment of lexical access of both nouns and verbs. This user-friendly application, allowing to accurately record scores and time responses and to get automatically the percentiles computed from the present normative data, holds the potential to become a popular tool. In particular, it provides the opportunity to easily measure the speed of lexical access, which correlates with return to work after glioma surgery [24].

The data were analyzed both at the item- and task-levels. At the item-level, all items had a name agreement > 80%, and available psycholinguistic variables were controlled between nouns and verbs.

Except for one verb, average response times were lower than 4 s, making this extraoperative set suitable for intraoperative mapping.

Significant differences in average scores and response times were observed between the two tasks: compared to the action naming task, the average score in the object naming task was slightly higher, and the average total response time 18 s shorter. Differences between nouns and verbs production have been the topic of numerous studies [1, 15, 32, 36] proposing several hypotheses to explain this dissociation. On the one hand, the lexical-semantic organization would be different: the nouns would be organized hierarchically (with a level of subordination between the categories: animals > mammals > felines > cat) while the verbs would have a matrix organization (classified in semantic fields such as verbs of movement, possession, vision, classified in turn based on other elements such as direction, the presence or absence of an instrument, etc.). On the other hand, verbs are by nature more grammatically complex, more abstract and less imageable than nouns (Rofes & Miceli, 2014), and their production therefore likely requires more cognitive effort than that of nouns. It was therefore expected that the action naming task would be a little bit less accurate and slower than the object naming task, as in Luzzatti's study [15] where the control population obtained a significantly lower naming score for verbs than for nouns. With regard to response times, the three studies by Bogka et al. [1] also showed a significant difference between nouns and verbs. However, this difference was no longer significant when the imageability and visual complexity of the verb images were controlled. Although these results must be put into perspective because of the small sample sizes in the above-mentioned studies, this hypothesis cannot be ruled out (see limitation section).

Gender had no influence on performances in the MULTIMAP, which is consistent with the literature: no effect of gender was found on oral naming performance in the Test de Dénomination de Québec-30 [16], the Batterie d’Evaluation Cognitive du Langage [17], or the Batterie d’Evaluation des Connaissances Sémantiques [22]. A minor effect of gender was noted in the Batterie d’Evaluation des Troubles Lexicaux [39] on scores only, not on reaction time, as well as in the Boston Naming Test [23].

Age, on the other hand, had a significant effect on scores and response times in the two MULTIMAP tasks: older subjects made more errors and were slower for both nouns and verbs. This age effect is found in all studies analyzing the effect of demographic characteristics on picture naming performance. Older subjects tend to correctly name fewer items with longer response times than younger subjects [16, 17, 22, 23, 29, 39]. No difference in performance between the two age groups of subjects under 50 years of age has also been found in the standardization of the Batterie d'Evaluation des Troubles Lexicaux [39].

A significant effect of educational level on subject performance was observed in the action naming test, where subjects without the French baccalauréat obtained a lower score and a longer response time than subjects with a level of education equal to or higher than the French baccalauréat. These results are consistent with those of a standardization study on an Italian action naming task, in which subject’s performances increased with their level of education [29]. Conversely, no effect of level of education was found in the noun naming test. This result may be related to the dissociation found in the literature between the production of nouns and that of verbs, which is thought to be more complex in terms of syntax and abstraction [36], which could explain the influence of educational level.

Given the statistical analysis of the effects of gender, age, and education, the score and time norms of the MULTIMAP therefore include three groups according to age for object naming task, and six groups taking into account both age and level of education for action naming task. The complete normative data for scores and response times includes for each group: the mean, standard deviation, minimum, maximum, and percentile distribution of performance. As the data did not follow a normal distribution, the use of percentiles to estimate subjects' performance seems preferable to a mean and a Z-score. Furthermore, the diagnostic and comparative purpose of the Multimap justifies the use of percentiles rather than a threshold score. As the differences in scores between the percentiles were small, it appeared sufficient to detail the 5, 10, 25, 50, 75, 90, and 95 percentiles instead of a 10 by 10 scale. The pathological threshold for scores and response times therefore corresponds to the 5th percentile and the fragility threshold to the 10th percentile —corresponding to the lowest-performing 5% and 10% of the sample, respectively.

## Limitations and future directions

As the digitized French MULTIMAP was being developed on the HappyNeuron platform at the same time as our study, we encountered a number of bugs: exporting the data proved to be complex and had to be done in several stages, and, for a couple of subjects, the alternative responses reported by the experimenters were deleted. These problems did not affect the recording of scores and response times, the results of the subjects concerned were therefore retained in our analyses. Only the percentages of alternative responses for a few items and the resulting response times, may have been very slightly impacted.

Seven different experimenters administered the digitized French MULTIMAP, which enabled us to recruit a large control population, warranting greater reliability in the statistical analysis and establishment of normative data. As we did not keep track of the subjects excluded because not fulfilling the inclusion criteria, we could not draw a flow-chart of the selection process, thus preventing to estimate how much our sample is representative of the entire population. In order to standardize data collection, the experimenters were trained in administering and scoring the test, and the accuracy of some unexpected responses was discussed and decided by group consensus. Nevertheless, we cannot rule out a slight inter-judge bias, the effect of which is minimal given the size of our control population and the type of equipment used, namely a standardized digital test which automatically records responses and response times. Nor can we rule out the possibility that there may have been some scoring errors during the tests. Indeed, a digital test can sometimes lead to rushing errors (errors when selecting the button to validate or invalidate the response, pressing too quickly before the subject has had time to respond, etc.). Finally, it is possible that there were differences in the acceptance of responses with expansions (noun followed by an adjective, verb followed by a complement, etc.), which may have had a discrete impact on the percentages of correct alternative responses.

In the proposed protocol, objects naming was tested before action naming. We cannot rule out that this serial (rather than intermixed) design might have partly explain the slightly lower score on verbs compared to nouns, because of attentional decrease during the task. Another limitation comes from the fact that we did not develop a second version, with entirely distinct pictures. Thus, for longitudinal follow-up, the task is subject to the test–retest effect. Lastly, it remains unknown how subjects with color blindness would perform the task, as colors might constitute an important cue for some items.

Additional psycholinguistics variables (like imageability, concreteness, age of acquisition, …) were not considered in the present work. The major reason is that we could not find any database providing these values for French verbs. Future studies could aim to fill this gap and to check if these variables are balanced between the 40 nouns and 40 verbs. Another limitation comes from the fact subjects were not asked if they were able to speak other languages than French. Including bilingual patients might have increased time responses.

Our study should be continued with clinical validation in neurological and neurosurgical patients. Patients' results on the French version of the digitized MULTIMAP could be compared with the results of tests commonly used to assess semantic access and lexical retrieval in France, such as the DO 80 or the Batterie d'Evaluation des Troubles Lexicaux.

## Conclusion

This study enabled the standardization of the digitized French version of MULTIMAP, a picture naming test composed of 80 color images (40 objects and 40 actions) specifically designed for longitudinal assessments in pre- and postoperative settings. Statistical analyses from a large control population (416 subjects aged between 18 and 95) showed that performance in the object naming task was influenced by age, while both age and educational level affected scores and response times in the action naming task. A validation study in patients undergoing awake-glioma surgery will be the next step of our research. With features such as automatic recording of responses and reaction times, the digitized French MULTIMAP should allow for more accurate longitudinal follow-up. It holds strong potential to be adapted in other languages, and thus to become a popular app for picture naming assessment.

## Supplementary Information

Below is the link to the electronic supplementary material.

ESM 1 Supplementary Material 1 (DOCX 26.0 KB)

ESM 2 Supplementary Material 2 (DOCX 18.7 KB)

ESM 3 Supplementary Material 3 (DOCX 18.1 KB)

ESM 4 Supplementary Material 4 (DOCX 18.1 KB)

ESM 5 Supplementary Material 5 (DOCX 19.0 KB)

## Data Availability

The data that support the findings of this study are available from the corresponding author upon reasonable request.

## Abbreviations

DO80: Dénomination orale de 80 images (80 pictures naming task)
Bac: Baccalauréat (high school diploma)

## Code availability

Data are available upon request to corresponding author.
