# Supplementary material for: Digital MULTIMAP: a standardization of objects and actions naming task in a french population
Source: Acta Neurochir (Wien). 2026 Jun 4;168(1):179. doi: 10.1007/s00701-026-06927-y (PMC13427860; doi:10.1007/s00701-026-06927-y)
Supplement: Supplementary file 1 — Supplementary Material 1 (DOCX 26.0 KB) [file 701_2026_6927_MOESM1_ESM.docx]

**Supplementary Table 1**

*Success Rate and Average Time per item for the 50 Objects and 50 Actions Tested*

| Nouns | | | | Verbs | | | |
| --- | --- | --- | --- | --- | --- | --- | --- |
| French | English | Success rate (%) | Average time (s) | French | English | Success rate (%) | Average time (s) |
| serviette | towel | 55,53 | 3,24 | imaginer | imagine | 1,92 | 2,484 |
| désert | desert | 60,34 | 3,806 | confesser | confess | 22,84 | 4,123 |
| bière | beer | 67,79 | 2,729 | accélérer | accelerate | 29,09 | 8,665 |
| mouche | fly | 71,88 | 2,876 | livrer | deliver | 42,79 | 4,143 |
| berceau | cradle | 76,2 | 2,388 | traverser | cross | 44,47 | 3,184 |
| cadeau | present | 78,13 | 2,462 | aider | help | 49,76 | 3,809 |
| béret | beret | 78,37 | 3,909 | transpirer | sweat | 50,24 | 4,515 |
| plage | beach | 81,97 | 2,808 | fermer | close | 56,25 | 2,897 |
| lune | moon | 82,21 | 3,103 | attendre | wait | 59,13 | 5,102 |
| dos | back | 84,13 | 2,541 | vendre | sell | 68,99 | 6,557 |
| manteau | coat | 84,13 | 2,254 | servir | serve | 74,04 | 3,466 |
| fleur | flower | 88,22 | 2,281 | gagner | win | 75,72 | 3,497 |
| beurre | butter | 89,9 | 2,347 | surligner | highlight | 76,68 | 3,352 |
| bouquet | bouquet | 90,63 | 2,174 | signer | sign | 80,53 | 2,552 |
| cuisine | kitchen | 90,87 | 2,482 | toucher | touch | 82,45 | 3,08 |
| jambe | leg | 90,87 | 2,562 | construire | build | 82,93 | 4,274 |
| épaule | shoulder | 91,59 | 2,55 | creuser | dig | 83,17 | 3,015 |
| glaçon | ice | 91,59 | 2,311 | saluer | greet | 83,17 | 3,319 |
| nœud | knot | 91,59 | 1,932 | poster | send | 83,41 | 2,818 |
| oignon | onion | 92,31 | 2,53 | étendre | hang | 83,65 | 2,86 |
| tasse | cup | 92,79 | 2,097 | ranger | place | 84,62 | 3,074 |
| bras | arm | 93,75 | 2,313 | se moucher | blow | 85,82 | 2,607 |
| queue | tail | 94,23 | 2,507 | nettoyer | clean | 87,74 | 2,492 |
| dent | tooth | 95,43 | 1,864 | chasser | hunt | 88,7 | 3,008 |
| cadenas | padlock | 96,15 | 2,388 | allumer | light | 89,66 | 3,235 |
| calendrier | calendar | 96,15 | 2,261 | casser | break | 89,9 | 3,131 |
| cochon | pig | 96,39 | 1,856 | brûler | burn | 91,83 | 2,666 |
| couteau | knife | 97,36 | 1,746 | monter | ascend | 92,31 | 2,245 |
| corde | rope | 97,6 | 2,162 | se marier | marry | 93,27 | 3,164 |
| château | castle | 98,08 | 2,082 | écouter | listen | 94,95 | 2,368 |
| cheval | horse | 98,32 | 1,947 | peindre | paint | 95,19 | 2,081 |
| chemise | shirt | 98,32 | 2,029 | peser | weigh | 95,91 | 3,48 |
| journal | newspaper | 98,32 | 2,163 | sauter | jump | 96,63 | 2,298 |
| œuf | egg | 98,56 | 1,812 | voler | steal | 97,12 | 2,541 |
| serrure | lock | 98,56 | 2,24 | prier | pray | 97,36 | 2,257 |
| fauteuil | armchair | 98,8 | 1,923 | s'asseoir | sit | 97,36 | 2,159 |
| poisson | fish | 98,8 | 2,039 | chanter | sing | 97,6 | 2,055 |
| chaise | chair | 99,04 | 1,758 | jouer | play | 97,6 | 2,432 |
| ciseaux | scissors | 99,04 | 2,074 | regarder | watch | 98,32 | 2,874 |
| écureuil | squirrel | 99,04 | 2,026 | mesurer | measure | 98,56 | 2,417 |
| lapin | rabbit | 99,04 | 1,872 | fumer | smoke | 98,8 | 2,107 |
| os | bone | 99,28 | 1,826 | découper | cut | 99,04 | 2,539 |
| vélo | bike | 99,28 | 1,847 | écrire | write | 99,04 | 2,265 |
| ceinture | belt | 99,52 | 1,777 | pousser | push | 99,04 | 2,123 |
| timbre | stamp | 99,52 | 1,819 | boire | drink | 99,28 | 1,925 |
| église | church | 99,76 | 1,829 | conduire | drive | 99,52 | 2,184 |
| feuille | leaf | 99,76 | 1,841 | courir | run | 99,76 | 1,96 |
| papillon | butterfly | 99,76 | 1,801 | lire | read | 99,76 | 2,038 |
| parapluie | umbrella | 99,76 | 1,89 | manger | eat | 99,76 | 2,181 |
| chapeau | hat | 100 | 1,712 | pêcher | fish | 99,76 | 2,399 |

*Note.* The items excluded in the final version are highlighted in red.
