# Supplementary material for: Digital MULTIMAP: a standardization of objects and actions naming task in a french population
Source: Acta Neurochir (Wien). 2026 Jun 4;168(1):179. doi: 10.1007/s00701-026-06927-y (PMC13427860; doi:10.1007/s00701-026-06927-y)
Supplement: Supplementary file 2 — Supplementary Material 2 (DOCX 18.7 KB) [file 701_2026_6927_MOESM2_ESM.docx]

**Supplementary Table 2**

*Alternative Responses Accepted for Eight Objects*

| Exact response | | Alternative responses | | Correct responses | |
| --- | --- | --- | --- | --- | --- |
| French  (English) | Rate (%) | French  (English) | Rate (%) | French  (English) | Rate (%) |
| Bière  (Beer) | 60.10 | Chope de bière  (Beer mug) | 7.69 | Bière, chope de bière  (Beer, beer mug) | 67.79 |
| Bouquet  (Bouquet) | 60.58 | Bouquet de fleurs  (Bouquet of flowers) | 30.05 | Bouquet, bouquet de fleurs  (Bouquet, bouquet of flowers) | 90.63 |
| Château  (Castle) | 79.33 | Château fort  (Fortified castle) | 18.75 | Château, château fort  (Castle, fortified castle) | 98.08 |
| Ciseaux  (Scissors) | 70.67 | Ciseau, paire de ciseaux  (Singular form of “ciseaux”, pair of scissors) | 28.37 | Ciseaux, ciseau, paire de ciseaux  (Scissors, singular form of “ciseaux”, pair of scissors) | 99.04 |
| Queue  (Nail) | 85.98 | Queue de chat  (Cat’s nail) | 8.25 | Queue, queue de chat  (Nail, cat’s nail) | 94.23 |
| Serrure  (Lock) | 94.23 | Trou de serrure  (Keyhole) | 4.33 | Serrure, trou de serrure  (Lock, keyhole) | 98.56 |
| Tasse  (Cup) | 80.77 | Mug  (Mug) | 12.02 | Tasse, mug  (Cup, mug) | 92.79 |
| Timbre  (Stamp) | 98.32 | Timbre-poste  (Postage-stamp) | 1.2 | Timbre, timbre-poste  (Stamp, postage-stamp) | 99.52 |
