# Supplementary material for: Digital MULTIMAP: a standardization of objects and actions naming task in a french population
Source: Acta Neurochir (Wien). 2026 Jun 4;168(1):179. doi: 10.1007/s00701-026-06927-y (PMC13427860; doi:10.1007/s00701-026-06927-y)
Supplement: Supplementary file 3 — Supplementary Material 3 (DOCX 18.1 KB) [file 701_2026_6927_MOESM3_ESM.docx]

**Supplementary Table 3**

*Alternative Responses Accepted for Seven Actions*

| Exact response | | Alternative responses | | Correct responses | |
| --- | --- | --- | --- | --- | --- |
| French  (English) | Rate (%) | French  (English) | Rate (%) | French  (English) | Rate (%) |
| Regarder  (Watch out) | 88.7 | Regarder la télé  (Watch TV) | 9.62 | Regarder, regarder la télé  (Watch, watch TV) | 98.32 |
| Etendre  (Hang out) | 80.53 | Etendre/tendre le linge  (Hang out/hang up the laundry) | 3.12 | Etendre, étendre le linge, tendre le linge  (Hang out, hang out/hang up the laundry) | 83.65 |
| Surligner  (Highlight) | 70.19 | Stabiloter, stabilobosser  (Verbs derived from a brand of highlighter pens) | 6.49 | Surligner, Stabiloter, stabilobosser  (Highlight, verbs derived from a brand) | 76.68 |
| Découper  (Cut out) | 65.38 | Couper  (Cut) | 33.66 | Découper, couper  (Cut out, cut) | 99.04 |
| Servir  (Serve) | 61.06 | Verser, verser à boire, servir à boire  (Pour, pour a drink, serve a drink) | 12.98 | Servir, verser, verser à boire, servir à boire  (Serve,pour, pour a drink, serve a drink) | 74.04 |
| Se moucher  (Blow one’s nose) | 83.65 | Moucher  (Blow) | 2.17 | Se moucher, moucher  (Blow one’s nose, blow) | 85.82 |
| Gagner  (Win) | 73.56 | Avoir gagné  (Have won) | 2.16 | Gagner, avoir gagné  (Win, Have won) | 75.72 |
