# Supplementary material for: Digital MULTIMAP: a standardization of objects and actions naming task in a french population
Source: Acta Neurochir (Wien). 2026 Jun 4;168(1):179. doi: 10.1007/s00701-026-06927-y (PMC13427860; doi:10.1007/s00701-026-06927-y)
Supplement: Supplementary file 4 — Supplementary Material 4 (DOCX 18.1 KB) [file 701_2026_6927_MOESM4_ESM.docx]

**Supplementary Table 4**

*Normative Data for the Objects Naming Task*

|  | **Score** | | | **Time (seconds)** | | |
| --- | --- | --- | --- | --- | --- | --- |
| Total  *n=416* | Age 18-49  *n=207* | Age 50-69  *n=109* | Age ≥70  *n=100* | Age 18-49  *n=207* | Age 50-69  *n=109* | Age ≥70  *n=100* |
| Mean | 38.1 | 37 | 35.9 | 80.3 | 88.8 | 102.4 |
| SD | 1.59 | 2.06 | 2.19 | 14.09 | 18.95 | 24.16 |
| Min | 32 | 29 | 30 | 56 | 57.7 | 68.9 |
| Max | 40 | 40 | 40 | 133.4 | 194.1 | 188.5 |
| P5 | 35.0 | 34.0 | 32.0 | 109.1 | 118.3 | 151.9 |
| P10 | 36.0 | 34.8 | 33.0 | 98.4 | 104.3 | 140.4 |
| P25 | 37.0 | 36.0 | 34.75 | 87.7 | 93.6 | 113.9 |
| P50 | 38.0 | 37.0 | 36.0 | 77.3 | 85.2 | 94.3 |
| P75 | 39.0 | 39.0 | 37.0 | 70.7 | 78.9 | 85.6 |
| P90 | 40.0 | 39.0 | 38.0 | 64.8 | 72.7 | 79.9 |
| P95 | 40.0 | 40.0 | 39.05 | 61.9 | 69.1 | 75.3 |

*Note.* n =number ; SD = Standard Deviation; Min. = Minimum ; Max. = Maximum ; P = Percentile.
