# Supplementary material for: Digital MULTIMAP: a standardization of objects and actions naming task in a french population
Source: Acta Neurochir (Wien). 2026 Jun 4;168(1):179. doi: 10.1007/s00701-026-06927-y (PMC13427860; doi:10.1007/s00701-026-06927-y)
Supplement: Supplementary file 5 — Supplementary Material 5 (DOCX 19.0 KB) [file 701_2026_6927_MOESM5_ESM.docx]

**Supplementary Table 5**

*Normative data for the Actions Naming Task*

|  | **Score** | | | | | | **Time (seconds)** | | | | | | |
| --- | --- | --- | --- | --- | --- | --- | --- | --- | --- | --- | --- | --- | --- |
|  | Age 18-49 | | Age 50-69 | | Age ≥70 | | Age 18-49 | | Age 50-69 | | Age ≥70 | |  |
| Total  n=416 | < Bac  n=62 | ≥ Bac  n=145 | < Bac  n=40 | ≥ Bac  n=69 | < Bac  n=32 | ≥ Bac  n=68 | < Bac  n=62 | ≥ Bac  n=145 | < Bac  n=40 | ≥ Bac  n=69 | < Bac  n=32 | ≥ Bac  n=68 |  |
| Mean | 37.0 | 37.8 | 35.8 | 36.9 | 33.8 | 35.8 | 98.6 | 94.1 | 112 | 106 | 132 | 125 |  |
| SD | 2.05 | 1.63 | 2.71 | 2.00 | 2.49 | 2.16 | 17.7 | 20.1 | 27.6 | 21.8 | 30.5 | 30.7 |  |
| Min | 31 | 32 | 27 | 29 | 29 | 31 | 69.1 | 62.3 | 72.2 | 74.5 | 79.7 | 85.7 |  |
| Max | 40 | 40 | 40 | 40 | 38 | 40 | 152 | 179 | 210 | 220 | 185 | 214 |  |
| P5 | 34.0 | 35.0 | 30.9 | 33.4 | 30.5 | 32.0 | 131 | 129 | 156 | 141 | 184 | 192 |  |
| P10 | 34.1 | 36.0 | 33.0 | 34.0 | 31.0 | 33.0 | 119 | 120 | 136 | 127 | 168 | 167 |  |
| P25 | 36.0 | 37.0 | 34.8 | 36.0 | 32.0 | 34.0 | 110 | 100 | 123 | 117 | 157 | 138 |  |
| P50 | 37.0 | 38.0 | 36.0 | 37.0 | 34.0 | 36.0 | 94.9 | 89.3 | 109 | 103 | 129 | 121 |  |
| P75 | 38.0 | 39.0 | 37.3 | 38.0 | 36.0 | 37.0 | 87.1 | 81.6 | 94.3 | 91.5 | 108 | 105 |  |
| P90 | 39.0 | 40.0 | 38.1 | 39.0 | 37.0 | 38.3 | 79.5 | 76.8 | 82.2 | 85.7 | 94.6 | 95.4 |  |
| P95 | 40.0 | 40.0 | 39.0 | 39.0 | 37.5 | 39.0 | 75.4 | 70.4 | 78.4 | 79.7 | 92.1 | 89.8 |  |
| *Note.* Bac= baccalauréat, French High School Diploma; n =number ; SD = standard deviation; Min = minimum ; Max = maximum ; P = percentile. | | | | | | | | | | | | |  |
